# Towards a More Particularist View of Rights’ Stringency

**DOI:** 10.1007/s11158-018-9396-3

**Published:** 2018-03-21

**Authors:** Benedict Rumbold

**Affiliations:** 1 Department of Philosophy, University College London, Gower Street, London WC1E 6BT, UK

**Keywords:** Rights, Stringency, Trumps, Particularism, Dworkin, Raz, Nozick

## Abstract

For all their various disagreements, one point upon which rights theorists often agree is that it is simply part of the nature of rights that they tend to override, outweigh or exclude competing considerations in moral reasoning, that they have ‘peremptory force’ (Raz in *The Morality of Freedom*, Oxford University Press, Oxford, 1986, p. 192), making ‘powerful demands’ that can only be overridden in ‘exceptional circumstances’ (Miller, in Cruft, Liao, Renzo (eds), *Philosophical Foundations of Human Rights*, Oxford University Press, Oxford, 2016, p. 240). In this article I challenge this thought. My aim here is not to prove that the traditional view of rights’ stringency is necessarily false, nor even that we have no good reason to believe it is true. Rather, my aim is only to show that we have good reason to think that the foundation of the traditional position is less stable than we might have otherwise supposed and that an alternative conception of rights—one which takes the stringency of any given right as particular to the kind of right it is—is both viable and attractive. In short, to begin to move us towards a more ‘particularist’ conception of rights’ standing in moral reasoning and judgement.

## Introduction

How stringent are rights? That is, at what point do they yield to competing considerations in moral reasoning and judgement (Edmundson 2004, p. 129)? Whatever writers’ various responses to this question, one point upon which almost all agree is that rights do not yield to much and there are at least some considerations to which they never yield. And this is for a simple reason: namely, it is just part of the nature of rights, *qua* rights, that they override, outweigh or exclude competing considerations in moral reasoning, that they have ‘peremptory force’ (Raz 1986, p. 192), making ‘powerful demands’ that can only be overridden in ‘exceptional circumstances’ (Miller 2016, p. 240).

In this article, I put forward an alternative account of rights’ standing, one which I call a ‘particularist’ account. According to this view, the decisiveness of any given right over any given set of competing considerations is not taken as something that follows from its status *as* a right—which is to say, a moral consideration of a certain kind—but rather is something particular to the kind of right it is, with one possibility being that it fails to present decisive reasons for action with respect to any competing consideration whatsoever.^1^

I motivate this position in three ways. First, I claim that there are significant problems in several of the main arguments by which writers have sought to defend the traditional view of rights’ stringency. Second—and relatedly—I suggest that an alternative conception of rights’ stringency is at least viable, or logically possible. Third, I argue that there are important advantages to recognising the possibility of at least some rights which routinely yield to alternative considerations in moral reasoning and judgement—and thus moving towards a more particularist view.

In presenting these arguments, my aim is not to show that the traditional view of rights’ stringency is necessarily false. Nor, indeed, is my aim to show that we have no good reason to believe the traditional view of rights’ stringency is true. (To prove *that*, one would have to show that every justification that has ever been offered of the traditional view is false, something which it is certainly beyond the scope of the present article.) Instead, my aim is only to show that the foundation of the traditional position is perhaps less stable than we might have otherwise supposed, and that an alternative conception is both viable and attractive. In short, to move us ‘towards’ a particularist conception of rights’ stringency, rather than present a definitive argument as to why our present position is unviable. Before getting into the main course of the discussion, however, let me say a bit more about the traditional view and the main claims about rights’ stringency I want to contest.

## Stringency

As set out above, the primary purpose of this paper is to challenge the notion that rights, *qua* rights, tend to override, outweigh or exclude other competing considerations in moral reasoning and judgement. This view makes two connected claims about rights’ standing: first, that there is something we can know about the stringency of any given right simply by virtue of knowing it is a right; second, that the *thing* one can know about the stringency of a right, simply by virtue of knowing it is a right, is that it is stringent to at least some extent. In what follows, I refer to the former as the generalist’s ‘abstract claim’ about rights’ stringency and the latter as the generalist’s ‘concrete claim’ (the labelling of such claims as ‘generalist’ being an indication that they are meant to hold as a general principle, which is to say, across all cases in which rights feature). I shall say a little bit more about these claims in a moment. However, first let me say something about what I mean by the claim that rights may be more or less ‘stringent’.

As I understand it here, to make a claim about the stringency of a given right is to make a claim about the *range* of moral considerations that right overrides, or outweighs, or excludes in moral reasoning and judgement; or, in my preferred way of putting things, the reasons in respect to which a right offers a *decisive* or *conclusive* reason for action.^2^ By a ‘conclusive reason’ here, I mean a reason for action which is able, by itself, to establish what we ought to do overall, or all things considered. In this, conclusive reasons may be properly contrasted with contributory reasons. A contributory reason, by contrast, is a reason that establishes what we ought to do absent any other reason, or all other things being equal, but which does not necessarily tell us what we ought to do overall, or all things considered (Dancy 2004, p. 16).

One important assumption of this article is that rights comprise one species of moral consideration, with other species including ‘social’ or ‘collective’ goals (such as the maximisation of happiness across a community) and ‘imperfect’ or ‘unipolar’ duties (such as duties of beneficence, or duties of honesty). Moreover, as I understand it, *as* one species of moral consideration, rights—like social goals, and imperfect duties—necessarily present us with *contributory* reasons for action. Which is to say, they present us with a case for acting in a certain way, all other things being equal. Insofar as we take rights as the *source* of certain ‘directed’ or ‘bipolar’ duties (cf. Tasioulas 2010, p. 656), or as endowing the right-holder with justifiable claims on others’ actions (Hart 1955), one natural way to understand the content of these reasons would be in terms of those duties generated by P’s right, each of which may be taken to describe how we ought to act with regards to the right-holder. (Note: by duties here, and in what follows, I mean a *pro tanto* moral requirement, which is to say, something which, all other things being equal, we morally ought to do or—as I take to be equivalent—it would be morally wrong not to do. In this, my use of the term duty differs from that of a number of other writers, who tend to use the term duty to denote a more binding set of obligations. Perhaps the most notable among these, in light of our present discussion, is Joseph Raz, for whom the word duty implies something it would *always* be wrong not to do, duties having ‘pre-emptive force’, ‘replacing’, rather than ‘competing with’, other reasons which apply in the circumstances (Raz 1986, p. 186)).

Assuming, then, that rights necessarily present us with contributory reasons for action, there is now a further question about whether, on occasion, rights might also present us with decisive reasons to act, or reasons that establish what we ought to do, all things considered. And it is with respect to this question that we are led to a discussion of rights’ ‘stringency’.

One view we might take here is that rights always present us with decisive reasons for action, regardless of whatever countervailing reasons are at play. To hold this position would be to endorse what is often referred to as an ‘absolutist’ conception of rights: namely, one which holds that there are no possible moral considerations in virtue of which a right may be overridden or justifiably infringed (any failure to fulfil a right effectively amounting to a violation, or unjustified infringement—cf. Gewirth 1986; McConnell 2000, p. 5).^3^ More temperately, however, we might think that the decisiveness of a right depends upon what kind of other moral considerations are arrayed against it, their number and make-up. For example, we might think that the fact that φ-ing infringes P’s right might constitute a decisive reason *not* to φ when judged in light of the reasons for action presented by moral considerations x, y and z, but it might not if one were to judge it in light of the reasons for action presented by moral considerations a, b and c. On this model, then, the stringency of a right might be seen to articulate a point along a range of possibilities, with rights being ‘more’ or ‘less’ stringent depending on which alternative moral considerations they are being held in judgement against, their number and arrangement.^4^ Thus, a maximally stringent right is one which is decisive regardless of whatever alternative moral considerations, or combinations of considerations are arrayed against it (being effectively ‘absolute’). A minimally stringent right is one which presents a conclusive reason for action in respect to only one other moral consideration. Finally, insofar as a right is not stringent at all, it only ever presents a contributory reason for action.

If this helps us understand what is meant when we talk about a right being more or less stringent, what sense can we make of the generalist’s abstract and concrete claims? Let us start with the abstract claim. One way we might understand the generalist’s abstract claim is as a claim about the relationship between the *stringency* of any given right and its status *as* a right, which is to say, a moral consideration of a particular sort. Specifically, what the generalist wants to say is both that there is a general rule about the stringency of rights that holds across all rights *and* that this follows, in some sense or other, from the fact that it is a right. To hold the generalist’s abstract claim, then, is not necessarily to hold any particular position about how stringent rights actually are. Rather, it is only to assert that we can know something about the stringency *of* a right simply by knowing that it *is* a right.

In this way, generalists who only endorse the generalist’s abstract claim may be seen to stand above debates about the extent of rights’ stringency. However, the kind of generalists I take myself to be addressing in this article also typically endorse another claim about rights’ stringency, one which *does* make a substantive claim about the extent of rights’ stringency. This is what I call the generalists’s *concrete* claim. According to this claim, then, it is not only that we can know ‘something’ about the stringency of a right simply by virtue of knowing it is a right, rather, we can also know that it is stringent *to at least some degree*, which is to say, *that it presents decisive reasons for action against at least some set of alternative moral considerations*.^5^

## Decisiveness

In a moment, I want to explore some of the ways in which generalists might defend the abstract and concrete claims. However, before doing so, there is one issue worth putting to rest: namely, how the present discussion about rights’ stringency, *qua* rights relates to a parallel debate about the way in which rights present decisive reasons for action, when they do.

Two models dominate the latter exchange: the ‘force’ model and the ‘exclusionary’ model. On a ‘force’ model, the interplay of rights with other moral reasons in matters of moral calculus is best understood as a kind of balancing act, wherein the reasons for action presented by rights, along with those presented with other moral reasons, have a certain ‘normative force’ or ‘weight’ which, when weighed together, tells us where our overall duty lies (cf. Lyons 1984, p. 113). In saying, then, that rights have a certain degree of stringency, that they present ‘decisive reasons for action’ when arrayed against some other set of moral considerations X, what we mean is that rights, *qua* rights, always necessarily ‘outweigh’ X (considered individually or collectively). In other words, were they to be deployed against such considerations (and only those considerations), rights would necessarily tip the balance of reasons in favour of the action they (the rights) demand—something they may or may not do with respect to other kinds of considerations, or when those considerations are joined by others. By contrast, on an ‘exclusionary’ conception, insofar as rights present decisive reasons for action against some other set of moral considerations, it is not by virtue of the fact that rights possess any greater force or weight than other reasons, rather it is that they *exclude* certain other kinds of reasons from playing any part in an overall judgement about what to do. As Raz—the originator of this theory—explains, we might easily get a grip on the notion of an ‘exclusionary reason’ by considering the case of a spouse who has promised their partner to decide how to spend the weekend in light of their own desires, irrespective of what they think their partner might want. In this case, ‘Their promises are exclusionary reasons, reasons to exclude a consideration from being the ground for any decision they may make’ (Raz 1988, p. 1158). Similarly, insofar as rights are decisive against another moral consideration, it is not that they offer reasons ‘one up’ on such considerations, or reasons of greater force, rather it is that they render such considerations ‘off the table’.^6^

How, then, does this debate bear on the present debate about the merits (or otherwise) of the generalist’s abstract and concrete claims? In one way, it has very little bearing. As described above, what interests the generalist, with regard to the abstract and concrete claim, is not the mechanisms by which a right presents decisive reasons for action in moral reasoning and judgement—that is, whether it is by virtue of the fact that rights’ possess greater normative ‘force’ or ‘weight’, or insofar as they exclude certain other reasons—but the relationship between rights’ decisiveness (however conceived) and their status as a right. In this way, then, generalists of the kind I intend to challenge here may stand above debates about how we ought to understand any decisiveness rights have, being amenable to both. This being said, however, it is worth noting that depending on which of the two models one endorses, what it means to *question* the generalist’s position will likewise differ. On the ‘force’ conception, then, what is being questioned here is the extent to which rights, *qua* rights, necessarily present reasons that outweigh at least some other reasons, as opposed to (sometimes) reasons that may be outweighed by others. On the ‘exclusionary’ conception, by contrast, what is being questioned is the extent to which rights, *qua* rights, necessarily present *reasons not to act for certain reasons* (i.e. exclusionary reasons), as opposed to (sometimes) simply *reasons to act in a certain way* (i.e. contributory reasons).

## Ontological Defences

Why endorse the generalist’s abstract or concrete claim about rights’ stringency? Since the generalist’s concrete claim is the more familiar of the two, let us start there. As set out above, the generalist’s concrete claim is the claim that, simply by virtue of knowing something is a right, we can know that it is stringent to at least some degree, which is to say, that it presents decisive reasons for action against at least some other moral considerations. Of course, what this leaves open is the question of *how* we can know, simply by virtue of something being a right, that it is stringent to at least some degree. One response might be to claim that it follows from the nature and logical properties of rights, which is to say, that it describes a feature rights must be held to possess *if they are to be understandable as rights*. Another response might be to claim that it describes a feature rights must be held to possess *if they are to be concepts worth recognising within our moral lexicon*. Then again, another response might be to claim that it describes a feature rights happen to possess *by virtue of their relationship to some overriding moral concern.*

In the hands of the generalist, each of these explanans become, in effect, justifications of the concrete claim. In what follows, then, I refer to the first of these as the generalist’s ‘ontological defence’ of the concrete claim, the second as the generalist’s ‘superficial defence’, and the third as the generalist’s ‘derivative defence’. In the rest of this section I consider various species of the ontological defence, before moving to the superficial defence and derivative defence in sections ‘Superficial Defences’ and ‘Derivative Defences’ respectively.

One way, then, in which one might motivate the generalist’s concrete claim is by arguing that it follows from the nature and logical properties of rights. There are a few ways this thought might go. First, one might argue that the claim that rights have a certain level of stringency necessarily follows from those characteristics that make a right a ‘right’ in the first place, which is to say, that *it is logically entailed by the properties that make rights ‘rights’*. Second, one might claim that such standing is not a property of rights logically entailed by those properties that make rights ‘rights’, rather *it is one of the properties that make rights ‘rights’*. Finally, one might argue that it is not one of the properties that make rights ‘rights’, rather *it is the only property that makes rights ‘rights’*; in other words, rights are *nothing other* than that set of moral considerations that present a decisive reason for action against other moral considerations. For ease of reference, I shall call the first of these the ‘weak’ ontological defence of the concrete claim, the second the ‘medium-strength’ ontological defence and the last the ‘strong’ ontological defence.

One thing these arguments bring to the fore, of course, is that, in some respects, our present discussion is as much a discussion about what constitutes the essential features of rights, *qua* rights, as it is about rights’ standing in matters of moral judgement and reasoning. This, in turn, raises questions about what constitutes proper grounds for asserting the merits of one conception of rights over any other. For example, as the arguments set out above intimate, one way proponents of the concrete claim might justify their position is by first asserting a conception of rights that renders the truth of the concrete claim effectively analytic. Thus, in line with the strong ontological defence, such an interlocutor might argue: ‘Rights are nothing other than moral considerations that present a decisive reason for action against other moral considerations. Therefore, simply by virtue of knowing something is a right, we can also know that it is stringent to at least some degree, which is to say, that it presents decisive reasons for action against at least some set of alternative moral considerations’.

If we are to challenge these arguments, then, we first need an independent set of criteria for evaluating what constitutes a satisfactory conception of rights. There look to be at least three desirderata. First, if a conception of rights is to meet a minimum standard of adequacy it needs to be logically coherent, which is to say, it cannot assert anything inconsistent or contradictory. Second, such a conception needs to demarcate rights as a distinctive concept in moral reasoning and judgement.^7^ Finally, conceptions of rights need to be able to make sense of the way we currently conceive of rights in moral, political and legal theory (cf. Raz 1986, pp. 165–166). A brief note on this last point: to say that such conceptions need to ‘make sense’ of the way we currently conceive of rights is not necessarily to claim that they need to mirror our preconceptions perfectly. After all, one of the purposes of this article is to suggest that at least some of our preconceptions about rights—or at least, about the way that rights function in moral reasoning and discourse—are mistaken. At the same time, however, we might agree that insofar as a given definition renders rights *unrecognisable*—which is to say, that it would fail to pick out the kind of concept we are referring to when we refer to P as having a right to φ—we might similarly think that whatever definition it is offering, it is not that of a right.

These desiderata accepted, it seems clear that the strong ontological defence does not offer a sufficient justification of the concrete claim. First, the conception of rights it relies upon—that rights are *nothing other* than moral considerations that present a decisive reason for action against other moral considerations—is too broad; it fails to meet the second desiderata. Assuming we think there are at least some moral considerations other than rights, rights cannot simply be moral considerations that present decisive reasons for action against other moral considerations, for this would imply that all moral considerations that present decisive reasons for action are necessarily rights, something which is clearly false. (Consider, for example, cases where no rights are present.) Second, relating to the third desiderata, the definition is also too circumspect; that is, it fails to pick out many of the characteristics that we tend to associate with rights, and to which we often appeal when attempting to differentiate them from other kinds of moral consideration.

A number of such features are widely accepted. (1) That rights denote a status of individuals or moral agents (a set which may be taken to include individuals other than human beings, corporations and/or groups—cf. Dworkin 2005, p. 91). (2) That rights seek to secure something that is good or valuable *for* that individual, something that it would be better, all things being equal, that they should have than not. In Raz’s view of rights, this is expressed in the thought that rights are grounded in individuals’ *interests*—see Raz (1986).^8^ (3) That rights engender ‘directed,’ or ‘relational,’ or ‘bipolar’ duties, which is to say, duties owed *to* the right-holder. (Hence, when we fail to abide by the reasons for action presented by an agent’s rights, we do not merely *do wrong*, rather we wrong *that agent*—cf. May 2015.) (4) That to fulfil an individual’s right is only to meet a minimal or required standard of moral behaviour, as opposed to doing anything supererogatory.^9^ All these features, then, are routinely recognised as features that help differentiate rights from other kinds of consideration (some necessary, others sufficient), yet they are all absent from the definition of rights as considerations that present decisive reasons for action.

Indeed, it is perhaps worth noting that this appears to be accepted even by those writers who might (wrongly) be read as claiming that rights are *nothing other* than moral considerations that present decisive reasons for action. For example, at times, Raz might be seen to endorse a conception of rights that holds them to be nothing other than decisive reason for action. As he puts it at one point ‘by definition rights are nothing but grounds of duties’ (Raz 1986, p. 176). (As above, note that by duties here, Raz means moral considerations that have ‘pre-emptive force’, which is to say, that ‘replace rather than compete with (some of) the other reasons which apply in the circumstances’—Raz 1986, p. 186.) However, it seems clear enough that Raz does not seek to define rights *solely* in respect of their moral standing. Rather, as he explains, his analysis of rights explains their ‘uniqueness’ as a ‘combination of two element’: (i) ‘rights have a special force which is expressed by the fact that they are grounds of duties, which are peremptory reasons for action’; (ii) ‘rights express what is owed to the right-holder in virtue of the respect due to his interest’ (Raz 1986, p. 249). Here (ii) appears to pick up (3) above.^10^

Insofar then, as we recognise that rights have features apart from their decisiveness in moral reasoning and judgement, it appears difficult to motivate the concrete claim by way of the strong ontological defence. What, though, of the medium-strength ontological defence? Might we not argue that rights’ imagined stringency with respect to other moral considerations is not the *only* property that makes rights ‘rights’ but that it is at least *one* of the properties that makes them ‘rights’? To make this claim, of course, one thing that we would need to show is that it is impossible to conceive of rights as rights without ascribing to them a certain stringency in respect to alternative moral considerations. In other words, that properties (1)–(4), say, are not, on their own, sufficient to meet our three desiderata. However, it is difficult to see how such an argument might go. That is, to my mind at least, the kind of properties gestured to in (1)–(4) look amply sufficient to meet our three desiderata of a satisfactory conception of rights without further appeal to any claim about rights standing in moral reasoning and judgement. In particular, in (3)—the claim that rights are moral statuses that engender directed or relational duties—we appear to have a perfectly understandable conception of what rights are, one which is logically coherent (desiderata one), that demarcates rights as a distinctive concept in moral reasoning and judgement (desiderata two) and that makes sense of the way we currently conceive of rights in moral, political and legal theory (desiderata three). To put this in Raz’s terms above: it seems perfectly possible to hold a conception of rights according to which ‘rights express what is owed to the right-holder in virtue of the respect due to his interest’ (ii), without needing to appeal to the further claim that ‘rights have a special force which is expressed by the fact that they are grounds of … peremptory reasons for action’ (i) (Raz 1986, p. 249).

Let us assume, then, that there is some set of properties we can ascribe to rights that provide us with a coherent conception of what rights are without necessarily including within that set the property that rights have a certain standing in moral reasoning and judgement. Still, might we not argue that such a standing is *logically entailed* by those properties that we *do* take to identify rights as rights (i.e. the weak ontological defence)? Again, if the properties in question here are those gestured to in (1)–(4) it seems difficult to see why this would be the case. For example, with respect to (3) it seems perfectly possible to assert that rights are the source of directed or relational duties without also saying anything about their stringency. This point is well made by Sreenivasan in respect to what he refers to as a Hohfeldian conception of moral claim-rights. As he explains, such a conception of rights is ‘clearly and importantly distinct’ from the notion of, say, rights as moral considerations that present decisive reasons for action, or what he terms ‘a deonological moral claim right’; and while nothing prevents Hohfeldian moral claim-rights from playing the role of its deonological counterpart, neither are they logically dependent (Sreenivasan 2010).

For these reasons, then, I take it that the generalist cannot defend the concrete claim via either the weak, medium-strength or strong onological defences. This is important to the current argument insofar as it undercuts one of the main set of arguments that could be used to defend the generalist’s position. However, it is also important in another way: namely, insofar as it helps us to see that an alternative conception of rights—that is one which accepts that they do not (always) present decisive reasons for action, or, as is the same, that they may, on occasion, only present contributory reasons for action—is at least *viable*.

What might this alternative conception of rights look like? And what kind of rights might it include? I will primarily deal with these questions in section ‘Rejecting Generalism’ However, to give an indication of what kind of conception we are working towards, it helps, perhaps, to consider the possibility of a right that fails to present decisive reasons for action against any competing considerations whatsoever. Consider the following case. In the British Library in London there is an informal arrangement whereby if one is the first to arrive at a desk in the morning, one may ‘reserve’ that desk by placing one’s books upon it. Books placed on the table, then, indicate that that desk is being used, even if no one is presently sitting there, and it also indicates that others ought not to use it. To my mind, this situation is best understood in terms of something like a property right. When I place my books on the table, I equally place you under an obligation not to move them, or otherwise use the desk. However, if this is a property right, then it also appears to be one that yields to almost any other moral consideration whatsoever. That is, it seems difficult to imagine any other consideration which could not constitute a good reason to move someone’s books.

Now, in suggesting this as one example of a right that is not stringent at all, one opens oneself up to numerous counters based on the case at hand. Does one’s temporary property rights over a desk space really constitute a *moral* right? And even if it does, are we sure there are no other moral considerations in respect to which it might not present a decisive reason for action? More pressing than these counters, perhaps, is a worry about whether it is really worth our time and energy to consider the moral status of these kinds of rights in the first place. ‘Is *that* what is at stake in all of this?’ one might ask: ‘We are discussing whether we ought to recognise temporary property rights to desk space as rights?’ However, this counter may be rebuffed in fairly short order. For, of course, what we are interested in here is not just the possibility of these kinds of rights but what we may legitimately say *about* rights, how we ought to understand them as moral objects and how we ought to understand the role that they play in moral reasoning and judgement. A right over a desk space, then, presents us with an example of (at best) a minimally stringent right but more important than that, it suggests that the possibility that there may be some rights we can coherently classify as rights, even though they do not (appear) to present decisive reasons for action (or at least, even if they do, *that* is not what makes them rights).

We have, though, moved slightly away from our discussion of the generalist’s position. Next, then, let us look at superficial defences.

## Superficial Defences

As set out above, along with the ontological defence, one way in which the generalist might seek to defend the concrete claim is by making what I have called the superficial defence. Unlike the ontological defence, to make this defence, the generalist need not insist on the idea that the concrete claim follows from the nature of rights as a moral concept in anything like a strict logical entailment. Instead, the generalist may claim that, although it may be possible for a right to be understandable as a right while failing to possess the standing demarcated by the concrete claim, for various reasons, the only rights worth recognising as rights are those that do possess such a standing.

One writer who might be read as making something like this argument is Ronald Dworkin in his ‘Rights as Trumps’ (Dworkin 1984). Now, it is not always entirely clear that Dworkin actually does intend to defend the concrete claim by way of the superficial defence as opposed to, say, the ontological defence. However, I think his arguments can certainly be read in this light, and, given their enormous influence in rights theory, they are certainly worth discussing here.

In ‘Rights as Trumps’, Dworkin argues that whatever our various disagreements about what rights *are*, we are justified in defining as rights only those rights that offer decisive reasons against collective goals (or, as he puts it, that ‘trump’ such considerations) because we have no need of rights *except* when we need to explain what would be wrong about endorsing a kind of ‘unrestricted’ utilitarianism. As he explains:

We need rights, as a distinct element in political theory, only when some decision that injures some people nevertheless finds prima-facie support in the claim that it will make the community as a whole better off on some plausible account of where the community’s general welfare lies. But the most natural source of any objection we might have to such a decision is that, in its concern with the welfare or prosperity or flourishing of people on the whole, or in the fulfilment of some interest, widespread within the community, the decision pays insufficient attention to its impact on the minority; and some appeal to equality seems a natural expression of an objection from that source. (Dworkin 1984, p. 166)

In this passage, then, Dworkin establishes a strong justification for why we might think we need rights as a concept in moral—as well as political—theory: namely that it enables us to account for wrong involved in decisions which ‘injure some people’ while simultaneously making the community as a whole better off. However, we need to be very careful here about precisely what Dworkin’s argument justifies. That is, while we might think that Dworkin’s argument gives us good reason to think we need *some* concept of rights in moral theory, it is not clear why we need a concept of rights as *decisive* reasons against collective goals. Why not think that rights merely show that there would be *something* wrong in ‘injuring some people’ in order that we might benefit the community; that is, that such injuries present contributory, rather than conclusive, reasons against some action?

One thing that Dworkin might say in response here, and indeed, something that he does say at certain points in his discussion, is that in certain situations we do not want to say that there is merely *something* wrong about such actions but rather that they are overall wrong, or all things considered wrong. As he puts it (seemingly appealing to the idea of something being overall wrong):

We want to say that the decision is wrong, in spite of its apparent merit, because it does not take the damage it causes to some into account in the right way and therefore does not treat these people as equals entitled to the same concern as others. (Dworkin 1984, p. 116)

Earlier in the essay he raises a good case in point here: namely, individuals’ rights to political independence. As Dworkin explains, one problem with endorsing utilitarianism as a general background justification for political decisions is that we (can) seem wedded to the thought that we must take into account each and everyone’s political (and/or moral) preferences, as well as their personal preferences, in our overall calculation as to the utility of a given state of affairs. We seem to need to count, for example, the Nazi’s preference that Jews are given less of a particular resource, or the homophobe’s preference to ban homosexuality. If we are to make utilitarianism an ‘attractive working political theory’, then, we need to ‘qualify’ it so as ‘to restrict the preferences that count by excluding [certain] political preferences’ (Dworkin 1984, p. 158). A right to political independence— that is, ‘the right that no one suffer disadvantage in the distribution of goods or opportunities on the ground that others think he should have less because of who he is or is not’—understood as a ‘trump’ over utilitarian concerns achieves this restriction by rendering inclusion of, and acting upon, such preferences as always, all things considered wrong (Dworkin 1984, p. 158). In so doing, then, Jews are ‘insulated’ from the preferences of the Nazis (Dworkin 1984, p. 158).^11^

Again, much of this seems right. Certainly we might agree that, in this case, Jews’ rights to political independence *do* ‘trump’, or offer decisive reason against, mere utilitarian concerns, and that we need a moral concept capable of performing this role. However, again, the mere fact we need to be able to assert that *some* rights present decisive reasons for action does not, in itself, suggest that we *only* need rights when they perform such a function. That is, why not think that rights continue to serve a useful function—that they are worth recognising as rights—when they fail to override the demands of some social goal; in short, when they only present a contributory reason for action?

Dworkin provides further arguments on this point in his earlier monograph, *Taking Rights Seriously*. As he explains, one of the reasons we *only* need rights when they have the kind of standing captured by the concrete claim is because there is simply ‘no point’ to recognising any right when it fails to meet that threshold. As he explains:

It follows from the definition of a right that it cannot be outweighed by all social goals….Suppose, for example, some man says he recognizes the right of free speech, but adds that free speech must yield whenever its exercise would inconvenience the public. He means, I take it, that he recognises the pervasive goal of collective welfare, and only such distribution of liberty of speech as that collective goal recommends in particular circumstances. His political position is exhausted by the collective goal; the putative right adds nothing and there is no point to recognising it as a right at all. (Dworkin 2005, p. 92)

I do not find this line of argument particularly persuasive. In the passage, Dworkin appears to want to argue that there is no need to recognise any right that failed to offer decisive reasons against collective goals—what we might call non-trumping or quasi-rights—because such rights ‘add nothing’ to moral reasoning. Yet this looks plainly false. That is, we might think that such rights play two significant roles within moral reasoning—and political theory for that matter—even where they fail to provide sufficient justification for overriding the demands of alternative moral considerations. First, simply by presenting a contributory reason for action, even if some quasi-rights necessarily bow to the demands of *all* other moral considerations, such rights would still be sufficient to determine what we ought to do, all things considered, when asserted on their own, which is to say, in absence of any other moral consideration. (All other things being equal, your right to the desk you have reserved still places me under an obligation not to use it, even if it is a duty that I might justifiably ignore in the face of any other moral considerations whatsoever.).

Second, by virtue of offering contributory reasons for action, even where, say, P’s quasi-right *does* fail to overrule, say, the demands of collective goal x, such rights still perform a vital role in our overall understanding of the moral facts insofar as they establish that *that* course of action—which is to say, the pursuit of collective goal x—while overall justified, is still one that comes at a moral cost: namely, its failure to fulfil P’s right. Thus, even if they fail to establish on their own what we should do all things considered, simply by favouring a certain course of action, quasi-rights still play an important function: they explain how an entirely justifiable moral decision may still be non-optimum, that it carries what Bernard Williams calls a moral ‘residue’ (Williams 1973, p. 179).^12^

A good case in point here is Feinberg’s well-known wilderness example:

Suppose that you are on a backpacking trip in the high mountain country when an unanticipated blizzard strikes the area with such ferocity that your life is imperilled. Fortunately, you stumble onto an unoccupied cabin, locked and boarded up for the winter, clearly somebody else’s private property. You smash in a window, enter, and huddle in a corner for three days until the storm abates. During this period you help yourself to your unknown benefactor’s food supply and burn his wooden furniture in the fireplace to keep warm. Surely you are justified in doing all these things, and yet you have infringed the clear rights of another person. (Feinberg 1980, p. 230)

Now the question of whether, as Feinberg claims, you *would* be justified in infringing the rights of the cabin holder in this situation—and therefore, that rights may be justifiably infringed—continues to be the subject of extensive debate (see e.g. Steiner 2013, p. 236). However, assuming, for the sake of argument, that the backpacker’s actions were justified, does the fact that, nevertheless, the cabin owner had a right to who used their cabin tell us anything about the moral situation? To my mind, it seem clear that it does: namely, it tells us that there is at least one consideration that tells against the backpacker’s actions; that, insofar as the backpacker infringes the cabin owner’s rights, even if their actions were overall justified, the cabin owner has at least some cause for complaint, perhaps even grounds for compensation.^13^ In other words, then, the moral situation would be quite different if the cabin was not owned by anyone.

Returning to Dworkin’s argument above, we can see that for similar reasons R’s endorsement of the view that, for all its moral significance, P’s right fails to trump collective goal x, does not imply that her political position is ‘exhausted’ by her endorsement of collective goal x; for any statement restricted to the claim ‘R endorses collective goal x’ while true, also fails to account for at least one facet of her position: namely her view that there are rights here at stake (albeit rights that can be justifiably infringed by collective goal x). There is still, then, a *point* to stipulating a right falling short of the kind of trumping threshold Dworkin posits, for recognition of such rights still helps us make sense of the moral features of the situation: i.e. that there is at least one consideration that counts against the pursuit collective goal x, and that consideration is the damage such a pursuit does to the right-bearer with respect to their right.

It seems difficult, therefore, to defend the concrete claim on the basis that there is no point to recognising those rights that fail to offer decisive reasons for action. At this stage, however, the generalist might be moved to make another argument in defence of the concrete claim, one which might also be bracketed under the banner of a superficial defence. That is, rather than arguing that it is ‘not worth recognising’ non-trumping or quasi-rights because such rights have no part to play in moral theory, the generalist might instead argue that it is ‘not worth recognising’ such rights in the sense that, were we to do so, we would lose the special status we have accorded them. Raz might be read as making this argument in defence of his own conception of rights as the grounds of duties. As he explains:

The idea of a right to personal autonomy, for example, is attractive partly because such a right establishes a limit to what can be demanded of an individual in the name of collective goals and of communal welfare. But if rights do not represent the special force of the interest of the right-holder then they cease to capture the idea of a protective shield against the claim of the well-being of others. (Raz 1986, pp. 249–250)

Again, I am not particularly persuaded by this line of argument (which it, ought to be said, has been voiced by numerous writers other than Raz—see e.g. Habermas 1999, p. 260; Young 2014, p. 48). According to Raz, we can ascribe a certain utility to reserving the name ‘right’ for only those rights that *do* present decisive reasons for action, for, by doing so, we know that, whenever we assert P’s right to u, we also know that we are talking about a moral object that presents a decisive reason for action. By contrast, were we to admit of some rights that failed to meet this criteria, it would be an open question as to whether P’s right to u constituted a decisive reason for action or merely a contributory one. However, while it is easy to see why it might be useful to restrict possible meanings of the term ‘right’ in this way, it also comes at a huge cost. By restricting our use of the term ‘right’ to only those rights that do present decisive reasons for action in moral reasoning and judgement, one makes it impossible to refer to any right that fails to present such reasons. Of course, insofar as Raz’s argument ultimately relies on an overall judgement about the costs and benefits associated with different uses of specific moral terminology, the extent to which we should leave the decisiveness of rights ‘open’ might be seen as something about which reasonable people might disagree. However, to my mind at least, it seems difficult to justify effectively removing a concept from our moral lexicon (i.e. that of a non-trumping or quasi-right) simply so that we know that whenever someone uses the term ‘right’ they are referring to a right that presents a decisive reason for action. If *that* is the price of such shorthand, then it seems too high.

It seems false, then, to claim, along with Dworkin, that there is no *benefit* to recognising as rights only those rights that are stringent to the extent demarcated by the concrete claim, or with Raz, that the *costs* of recognising such rights are greater than their benefits. Either way we read it, then, it looks like there are significant problems with a superficial defence of the concrete claim.

## Derivative Defences

Let us now turn to one last set of arguments that have been put forward in defence of the concrete claim: namely, derivative defences. As with the superficial defence, in making this argument, the generalist may accept that it is perfectly possible for some rights to fall short of the kind of moral standing prescribed by the concrete claim. Moreover, against the superficial defence, they may even accept that were such rights to exist they would be ‘worth recognising’. However, at this point the generalist may interject that the entire discussion of whether such ‘quasi-rights’ are possible, or valuable assets within our moral theory, is moot, for the only rights that exist are those that *do* possess such a standing. And this is because, so the argument goes, *all* rights derive from a moral principle of primary moral importance. Thus, we can know by virtue of something being a right that it is stringent to at least some extent (the concrete claim), by virtue of the fact that all rights derive from some overriding moral concern (the derivative defence).

One writer who might be read to have been making something like this argument is Robert Nozick. Famously, Nozick holds a view according to which rights act as ‘side-constraints’ upon our action: ‘don’t violate constraints C’ (Nozick 1974, p. 29). Nozick defends this view, in part, by appealing to the Kantian principle that individuals are ends and not merely means, something he interprets as the dictum ‘they may not be sacrificed or used for the achieving of other ends without their consent’ (Nozick 1974, p. 31). Later, he adds further content to this thought by claiming that *to* use a person (say, for the benefit of others), is to fail to ‘sufficiently respect and take account of the fact that he is a separate person’ (Nozick 1974, p. 33). Thus, in Nozick’s own summary of his position:

The moral side constraints upon what we may do, I claim, reflect the fact of our separate existences. They reflect the fact that no moral balancing act can take place among us; there is no moral outweighing of one of our lives by others so as to lead to a greater overall *social* good. There is no justified sacrifice of some of us for others. (Nozick 1974, p. 33)

One way, it seems, that we might characterise Nozick’s defence of the concrete claim is as resting on a moral view which accords lexical priority to individuals’ self-ownership over all other moral concerns (cf. Cohen 1995, p. 67; Arneson 2011, p. 23; although, for a note of caution about placing too much emphasis on this see Mack 2015). First among moral principles is the principle that in any dealings, we ought to respect individuals’ sovereignty over themselves as ‘distinct individuals each with his *own* life to live’ (Nozick 1974, p. 34). Since, then, all moral rights derive from this fundamental moral principle, and since this principle has lexical priority over any other, all moral rights have lexical priority over any other moral consideration; or, in other words, rights act as ‘moral side constraints upon what we may do’.

There are, however, a number of problems with this position. First, it seems difficult to motivate the notion that, among all the possible moral concerns we might have, individuals’ sovereignty over themselves has *lexical* priority, such that, even if we were to add all other moral concerns together, they would be insufficient to override it. The preservation of life on earth, for example, might be seen as something that might take precedence over the respect we ought to accord to an individual’s sovereignty over themselves. Second, it is also not clear why individuals’ rights, as opposed to any other moral concept, are particularly suited to protecting individuals’ self-ownership.^14^ Why not think that individuals’ sovereignty over themselves as ‘distinct individuals each with his *own* life to live’ is better protected through a system of imperfect or unipolar duties? And if they can be, why think that it is *rights* that act as side-constraints on what we may do, which may never be justifiably infringed, rather than, say, some set of unipolar duties?

Finally, and perhaps most importantly, even if we accept that some principle of individual self-ownership has priority over other moral concerns, and that rights are particularly suited to protecting such self-ownership, it is not entirely clear why we should think that there are no rights *other* than those that protect self-ownership. One case in point here is individuals’ property rights (that is, their rights to property over things other than themselves). It seems reasonable to suppose, for example, that individuals have moral rights over their property. However, at the same time, if individuals do have moral rights over their property, it does not seem to be in virtue of some claim about the moral significance of *self*-ownership. Which is to say, it does not (necessarily) seem to violate an individual’s rights of self-ownership to seize their *property*. It might, on Nozick’s line of thinking, be a violation of such a principle to seize *that person*, to use them as one would a tool, but not their *property*, that is, not the objects they possess. In what sense would that be using someone as a tool?^15^ If we accept, though, that people can have moral rights over their property and that such rights are not derived from individuals’ sovereignty over themselves, then it also looks like there may be some rights that do not derive from that moral principle. And if that is the case, then there seems little reason to think that all rights necessarily act as side-constraints upon action; or, that all rights, *qua* rights, are stringent to at least some extent (i.e. the concrete claim).

Of course, any generalist hoping to defend the concrete claim through the derivative defence need not rely on Nozick’s particular presentation of that argument (or the presentation of that argument we have ascribed to Nozick). However, the thought here is that whatever argument the generalist makes at this point they will face the same three difficulties: first, in establishing the lexical priority of whatever moral principle they take rights to derive from; second, in showing that rights are uniquely suited to protecting whatever that moral principle denotes as of value; and third, in showing that there are no rights worth recognising as rights that do not derive from that moral principle. It is this last obstacle that, to my mind, looks especially insurmountable for the generalist, for it seems that we might always find at least some rights that do not derive from a single moral principle. And yet, insofar as we do find such rights, one cannot use the derivative defence to justify the generalist’s concrete claim.

## The Abstract Claim

To review: thus far I have considered three kinds of defences of the generalist’s concrete claim—ontological defences, superficial defences and derivative defences. My presentation of these arguments in no way reflects the totality of those arguments that the generalist may employ. However, the question arises, if the generalist *were* willing to give up the concrete claim, might they still hold on to the *abstract* claim?

Recall, the abstract claim was the claim that however stringent one takes rights to be, one can say something about the *extent* of the stringency of any given right simply by knowing that it *is* a right. As discussed in section ‘Stringency’, the generalist is free to make this claim independently of the concrete claim as it has been laid out here. However, the problem for the generalists at this stage is that if they accept, as we have assumed they have done, that the ontological defence of the concrete claim is false, then their abstract claim also appears somewhat redundant. That is, if we accept that there is at least the possibility that rights may only provide contributory reasons for action, it also seems clear that there is no reason to think we can say anything about the extent of a right’s stringency simply by knowing that it is a right. Rather, the mere identification of a right as a right gives us little information on its stringency one way or the other: it may present decisive reasons for action, or only contributory reasons. Against the prescriptions of the generalist’s abstract claim, there is no general rule about the stringency of rights that follows from their status as rights.

## Rejecting Generalism

What, then, if we withdraw our support from the generalist’s claims entirely? What kind of conception of rights are we left with?

First, it helps to consider what denying the generalist’s abstract and concrete claims does *not* commit us to. For example, one thing that denying the traditional view of rights’ stringency does *not* commit one to is a ‘weak’ conception of rights, that is, one which takes rights to be routinely overridden by all other moral considerations. Of course, insofar as the present conception allows for the possibility of rights that only act as contributory reasons, it could be viewed as more friendly to such a conception than the generalist’s.^16^ However, what is being challenged here is not the notion that rights *have* such-and-such a level of stringency, but rather that this is something that can be said of them *qua* rights. Returning to the explication of the generalist’s abstract claim in section ‘Stringency’, the non-generalist may agree with the generalist that there is a general rule about the stringency of rights that holds across all rights. What they want to deny is the further claim that this is something that follows, in some sense or other, from the fact that it is a right.

Following on from this point, to recuse oneself from endorsing the generalist’s abstract and concrete claim does not also necessarily commit one to the idea that rights are *variable* in their stringency. That is, it may be (although it is unlikely) that when we look at each and every right, we find that they all possess a certain level of stringency, even that they are stringent to precisely the extent that Dworkin, or Raz, or others, attest. Rather, what the present account asserts is not that rights are necessarily *variable* in their stringency, but that a right may still be recognised as a right even if it fails to present decisive reasons for action with respect to at least some set of alternative moral considerations.

What emerges from all of this, then, is a more particularist take on rights’ stringency. What the denier of the generalist’s abstract and concrete claims wants to say—and, indeed, the only thing that they *have* to say—is that by virtue of the fact that some rights may only present contributory reasons for action, one cannot know the stringency of a given right just by virtue of knowing it is a right. For some readers this might come as something of a shock. However, it is worth stressing that in endorsing a particularist conception of rights’ stringency we are only bringing rights back into line with how we think about other kinds of moral consideration. That is, we are only asserting that the extent to which rights override other kinds of moral considerations is undetermined simply by virtue of their status as moral considerations of a particular sort.

As well as coming at little cost, it is also perhaps worth noting that the present model also has certain advantages. For example, as well as allowing for the possibility that there are *some* rights that are just as stringent as, say, Dworkin suggests, against Dworkin’s position, the present model is also able to acknowledge the existence of certain rights that fail to meet Dworkin’s ‘trumping’ threshold. That is, it is able to acknowledge the existence of what, for Dworkin, would be a ‘quasiright’. In this sense, then, the present model allows for a broader ecology of rights than one which we normally think possible, one which includes both those rights that present decisive reasons for action against rival moral considerations, and those that merely present contributory reasons for action.

Another benefit of the present model is that it provides argumentative resources for those who would defend rights against some of their critics. For example, one criticism that is often levelled at rights-talk is it tends to be overly ‘dogmatic’. As Glendon (2008, p. 14) puts it, insofar as rights tend toward ‘absoluteness’, they simultaneously ‘promote unrealistic expectation, heighten social conflict, and inhibit dialogue that might lead towards a consensus, accommodation, or at least the discovery of a common ground’. All this, however, is avoided when we recognise that there may be some rights that only present contributory reasons for action. For insofar as we do *that*, it no longer becomes the case that to talk about rights is necessarily to talk about ‘absolute’ moral considerations, or reasons for action that are decisive in all areas in which they occur, but instead to talk about a set of moral considerations that present us with reasons for action that may be more or less decisive, or not decisive at all.^17^

Finally, the present model serves an important epistemic function. That is, in establishing the possibility that some rights may only present contributory reasons for action, the present account eschews the thought that we can reach justified conclusions about what we ought to do, all things considered, simply by looking to the *type* of moral considerations at play. Rather, because we know that not all rights necessarily present decisive reasons for action in all cases in which they occur, when we are trying to reach judgements about what we ought to do, we need to to look again at the nature of the right at hand and how it functions with respect to other kinds of considerations.

## Particularism

Before concluding, I want to offer a brief final note about the description of this account as a ‘particularist’ one. In this article I have labelled the conception of rights’ stringency I have been developing a particularist account because, in denying the abstract and concrete claims about rights’ stringency, it denies two generalisations that are often held to be true of rights *qua* rights. In this sense, the stringency of a right is, on my conception, taken to be more ‘particularist’ than previous, more ‘generalist’ conceptions.

This being said, however, there are certain, even more particularist conceptions of rights available that some particularists may view as more deserving of the moniker ‘a particularist conception of rights’ than the conception that I have outlined here. For example, one generalisation about rights that I have not contested thus far is the notion that all rights at least present us with contributory reasons for acting *in the way required by the relevant right*. Yet there may be some particularists who want to deny even this general principle. According to this account, then, there may be some cases in which the presence of a right within a moral dilemma fails to present us with any reasons to act in the way required by that right. Indeed, it might even be that they present reasons for us to act in a way utterly at odds with what that right requires. The fact that my stealing your loaf of bread violates your property rights, then, might, in some contexts, fail to count as a consideration against that action, and may even count in its favour.

In one sense, to hold *this* position would be to endorse a more resolutely ‘particularist’ conception of rights than the one I have put forth here. One thing that this conception respects, for example, is the particularist dictum that reasons for action are entirely context-dependent, in the sense that they have the potential to shift valence from context to context (Dancy 2004). Personally, I find myself fairly sympathetic to this position. That is, it seems possible that there may be some cases in which, say, the fact that an action violates P’s property rights fails to count as a consideration against that action, and even counts as a consideration in favour of it.^18^ However, such positions rest on arguments that are largely outside the scope of this paper. In the interests of brevity, therefore, I have left the present argument as leading us simply towards a particularist account of *rights’ stringency*, rather than a particularist account of *rights*.

## Conclusion

For all their various disagreements, writers on the metaethics of rights often agree on two things: that we can say something about the stringency of a given right just by knowing that it is a right, and that the thing we can know about its stringency is that it is stringent to at least some extent. In this article, I have contested these claims. In so doing, I have also attempted to start to move us towards a more particularist conception of rights’ stringency, which is to say, one which takes the stringency of any given right as particular to the kind of right it is, rather than something that follows from its membership as a moral consideration of a particular sort. Such arguments are not intended to be conclusive. There are far too many possible justifications of the generalists’ position for one to conclude, on the basis of the limited range of arguments considered here, that it is entirely without merit. (Far too little has been said, for example, about Raz’s justification for his view of rights, and derivative defences other than Nozick’s.) However, at the same time, I hope to have shown that *some* of the bases of that position are perhaps less stable than we might otherwise think, that an alternative conception of rights is viable and, in certain respects, fairly attractive.

## Footnotes

1 There are those who have endorsed similar positions. Brandt, for example, sets out a conception of rights which takes all rights as ‘only prima facie, not only in the sense that they may be overridden by other rights in certain circumstances, but also that they may be overridden by other moral considerations that are not matters of rights at all’ (Brandt 1992, p. 190). Where I disagree with Brandt, however, is on the metaethical justification for such a position (my argument following from a common conception of what rights are and the work they do in moral reasoning, Brandt’s position being at least partly a product of his wider rule-utilitarianism). A much closer position to mine, albeit one that still diverges at certain points and one which is again motivated on quite different grounds, is Danny Frederick’s conception of ‘pro-tanto’ rights (Frederick 2014).

2 In this, I broadly follow Sreenivasan (2010), although there are differences to our accounts.

3 One writer to whom something like this position is often ascribed is Robert Nozick. In his *Anarchy, state, utopia*, for example, Nozick claims ‘the side-constraint view (i.e. his view of rights) forbids you to violate these moral constraints in the pursuit of your goals’ and ‘individuals are inviolable’, both of which appear absolutist in tone (Nozick 1974, p. 31). However, elsewhere Nozick appears to have recognised that there may be some moral considerations that would justify infringing an individual’s right—most notably in order to avoid ‘catastrophic moral horror’. Another writer who comes very close to an absolutist view is Hillel Steiner, who holds a strong version of what he calls the ‘Moral Primacy’ thesis about rights. Steiner attempts to avoid what might otherwise seem decisive counter-examples to this view through a version of specificationism, effectively arguing that in cases where it appears that a right maybe overridden or justifiably infringed, it is because we have often underspecified the putatively overridden rights (see e.g. Steiner 2013).

4 To this we might add Thomson’s insight that ‘It does not even seem to be obvious that there is any such thing as *the* degree of stringency of any given right. Perhaps a right may be more or less stringent, as the rightholder’s circumstances vary, and also, in the case of special rights, as the means by which he acquired the right vary.’ (Thomson 1981, p. 136). However, for the sake of convenience, I leave these complications to one side for the time being.

5 In fact, many of the writers I would call ‘generalists’ tend to think rights are actually far more stringent than the concrete claim suggests. Thus, on their account, it is not just that rights, *qua* rights, necessarily present decisive reasons for action against at least some set of alternative moral considerations, they present decisive reasons for action against a whole host of alternative moral considerations. However, since I take it that all the challenges I make to the generalist position in what follows hold even with respect to a minimalist species of that view, and since any critique of the minimalist position necessarily implicates the maximalist position (a maximalist about rights necessarily being committed to the minimalist position), we can feel confident about meeting the generalist on the most parsimonious description of her position.

6 For commentary see e.g. Edmundson (1993), Gans (1986), Moore (1988) and Perry (1988).

7 One thing that this desiderata assumes, of course, is that there are moral considerations other than rights. Some may question this. However, I take it that the standard view here is that there are moral considerations other than rights—most notably, ‘collective’ or ‘social’ goals and ‘imperfect’ or ‘non-directed’ or ‘unipolar’ duties—and that our definition of rights needs to explain how the role rights play in morality differs from that played by these kinds of concepts.

8 Kamm offers a forceful challenge to this kind of condition. As she explains, some rights may be justified by the object’s moral nature directly, rather than by the object’s interests: Persons might have a right to treatment as equals … without our duty to them being based on their interests. Rather, I would say, this right is based on their nature as persons and not necessarily related to any aspect of their well-being. Even if it turns out to be in their interest to have this nature, the right derives from their nature and not from their interest in having it. … If there were an independent ‘dignitary interest’ in being treated as an equal … it need not be because this treatment serves that interest that a person has a right to it. It may simply be fitting to treat a person no differently from anyone else. (Kamm 2007, p. 246) Unfortunately, there is not sufficient space here to enter into an extensive discussion on this point (see e.g. May 2015, for more on this). However, I take it that, even if, from Kamm’s perspective, (2)’s formulation as it is set out above might appear a little clunky, (i.e. insofar as it fails to respect the fact that a right maybe grounded simply in, say, a human being’s nature as a rational agent who can value and pursue ends of her own, rather than in something good for that agent), there are ways of formulating (2) that would be able to acknowledge the possibility of the kind of rights to which she refers.

9 Feinburg might be seen to be gesturing to something like this idea when he argues that, when rights are fulfilled, ‘there is no place for gratitude, an expression of which would suggest that it is not simply one’s own or one’s due that one was given. Both kinds of transaction are important, and any world with one but not the other would—in Wasserstrom’s phrase—be “morally impoverished.”’ (Feinberg 1966, p. 142).

10 A similar point may be made with respect to Dworkin. At one point, for example, Dworkin appears to define rights merely by the extent to which they present decisive reasons for action against collective goals, writing that individuals have rights only when, ‘for some reason, a collective goal is not a sufficient justification for denying them what they wish, as individuals, to have or to do, or not a sufficient justification for imposing some loss or injury upon them’ (Dworkin 2005, p. xi). However, elsewhere Dworkin recognises that rights possess characteristics outside their ‘force’ against ‘competing claims’ (Dworkin 2005, p. 91). For example, on Dworkin’s account, a right is ‘an individuated political aim’ (apparently picking up (1) and (2) above). As he explains: An individual has a right to some opportunity or resource or liberty if it counts in favour of a political decision that the decision is likely to advance or protect the state of affairs in which he enjoys the right, even where no other political aim is served and some political aim is disserved thereby, and counts against that decision that it will retard or endanger that state of affairs, even when some other political aim is thereby served. (Dworkin 2005, p. 91) In this, Dworkin takes rights to contrast with collective goals, where ‘a goal is a non-individuated political aim, that is, a state of affairs whose specification does not in this way call for any particular opportunity or resource or liberty for particular individuals’ (Dworkin 2005, p. 91).

11 At this point, Dworkin might be seen to be prevaricating between a conception of rights as forming a protective barrier around certain individual interests, and a conception of rights as refining utilitarian justifications by excluding certain types of preferences. For a discussion of how Dworkin employs these two types of conceptions and various problems involved his (apparent) endorsement of both, see Yowell (2007).

12 See also Thomson’s critique of specificationism in rights (Thomson 1990, pp. 82–104).

13 One of Steiner’s misgivings about the kind of model being suggested here is that it leaves the question of whether the cabin owner is owed compensation ‘open’ (Steiner 2013). However, it is open to defenders of the model I am suggesting to claim that *all* infringements of a person’s rights provide legitimate grounds for compensation, only that there may be some rights which are routinely justifiably overridden.

14 For an interesting discussion on this, see Mack (1981).

15 Nozick, it should be noted, attempts to prove the exact opposite. As he puts it: … patterned principles of distributive justice involve appropriating the actions of other persons. Seizing the results of someone’s labour is equivalent to seizing hours from him and directing him to carry on various activities. If people force you to do certain work, or unrewarded work, for a certain period of time, they decide what you are to do and what purposes your work is to serve apart from your decisions. This process whereby they take this decision from you makes them a *part-owner* of you; it gives them a property right in you. Just as having such partial control and power of a decision, by right, over an animal or inanimate object would be to have a property right in it. (Nozick 1974, p. 172) In this passage, then, Nozick appears to want to claim that violations of one’s property rights necessarily violate self-ownership, for insofar as some controlling party takes your property they are, in effect, ‘forcing’ you do extra work in service of your goals, and that by taking *this* decision from you (i.e. the decision *not* to do further work in service of your goals), they become a ‘*part-owner* of you’. However, there is little in this line of argument that is convincing. In particular, merely putting someone in a situation where they are ‘forced to work’, even ‘taking decisions from them’, looks a long way from ‘owning’ that person, in part or in whole. Yesterday I parked my car in the last spot available in the communal parking bay outside my house. In doing so, I ‘forced’ my neighbours to find another spot to park their car, taking their decision to park in the spot I used ‘from them’. I fail to see any way in which this makes me a ‘part-owner’ of them.

16 Although, it is worth noting that even in cases where we find rights that fail to present decisive reasons for action that is not to say such rights are meaningless, or that we may simply ignore the reasons for action they present. As noted in section ‘Stringency’, simply by virtue of being a right—which is to say, a moral consideration of a certain kind—rights necessarily present contributory reasons for action. If, then, such reasons are to be overridden, they must still be countermanded by contrary reasons of a certain kind. In Gewirth’s terms, if a moral consideration is to override P’s right, that infringement still needs to be a justifiable one, otherwise it is simply a violation (Gewirth 1986).

17 An additional line of criticism that might be seen as analogous to Glendon’s—and hence one which might also be seen as answered by the present model—is the feminist critique that seeks to identify some of the more peremptory and rigidifying discourses around rights with a confrontational masculine ‘voice’ (Gilligan 1993).

18 Say, for example, that we imagine that Robin Hood’s project of stealing from the rich to give to the poor was not only part of an effort to redistribute wealth in his society but also a way of confronting a certain class in that society—in a way they will find immediately arresting—with the injustice of a society that had grown overly protective of its property rights (at the cost of all other moral considerations). In such a situation, we might say that the fact that Robin Hood’s actions violate Prince John’s property rights do not constitute a reason *not* to steal his money but rather a reason *to* steal it. In other words, there would be even less reason to take the money if it were not *his*.
